# Multidrug-resistant bacteria compensate for the epistasis between resistances

**DOI:** 10.1371/journal.pbio.2001741

**Published:** 2017-04-18

**Authors:** Jorge Moura de Sousa, Roberto Balbontín, Paulo Durão, Isabel Gordo

**Affiliations:** Instituto Gulbenkian de Ciência, Oeiras, Portugal; Wageningen Universiteit en Researchcentrum

## Abstract

Mutations conferring resistance to antibiotics are typically costly in the absence of the drug, but bacteria can reduce this cost by acquiring compensatory mutations. Thus, the rate of acquisition of compensatory mutations and their effects are key for the maintenance and dissemination of antibiotic resistances. While compensation for single resistances has been extensively studied, compensatory evolution of multiresistant bacteria remains unexplored. Importantly, since resistance mutations often interact epistatically, compensation of multiresistant bacteria may significantly differ from that of single-resistant strains. We used experimental evolution, next-generation sequencing, in silico simulations, and genome editing to compare the compensatory process of a streptomycin and rifampicin double-resistant *Escherichia coli* with those of single-resistant clones. We demonstrate that low-fitness double-resistant bacteria compensate faster than single-resistant strains due to the acquisition of compensatory mutations with larger effects. Strikingly, we identified mutations that only compensate for double resistance, being neutral or deleterious in sensitive or single-resistant backgrounds. Moreover, we show that their beneficial effects strongly decrease or disappear in conditions where the epistatic interaction between resistance alleles is absent, demonstrating that these mutations compensate for the epistasis. In summary, our data indicate that epistatic interactions between antibiotic resistances, leading to large fitness costs, possibly open alternative paths for rapid compensatory evolution, thereby potentially stabilizing costly multiple resistances in bacterial populations.

## Author summary

Antibiotics target essential cellular functions, such as translation or cell wall biogenesis, and bacteria can become resistant to antibiotics by acquiring mutations in genes encoding those functions. This causes most drug-resistance mutations to be detrimental in the absence of the drug. However, bacteria can reduce this handicap by acquiring additional mutations, known as compensatory mutations. Compensatory evolution is crucial for the maintenance and dissemination of antibiotic resistances in bacterial populations. While compensation for single resistances has been extensively studied, compensatory evolution of multidrug-resistant bacteria remains unexplored. Importantly, interactions between resistance mutations are frequent, and this may cause compensation of multidrug-resistant bacteria to differ significantly from that of single-resistant strains. By comparing compensation of single- and double-drug–resistant *E*. *coli*, we found that double-drug–resistant bacteria compensate faster than single-drug–resistant strains. This is due to the acquisition of compensatory mutations with larger effects and possibly driven by the large fitness cost of double-drug resistance. Strikingly, we identified mutations that compensate specifically for the interaction between drug resistances, since they are beneficial only for double-drug–resistant bacteria and in conditions in which the interaction between resistances occurs. In summary, our data indicate that certain interactions between antibiotic-resistance mutations can open alternative paths for rapid compensatory evolution, thereby potentially stabilizing multiple drug resistances in bacterial populations.

## Introduction

Bacteria evolve to endure antibiotics through the acquisition of genes or chromosomal mutations that confer resistance to the drugs [1–3]. Resistance mutations are widespread in clinical [2,4,5] and environmental [6,7] bacterial populations, providing a reservoir that can be transmitted by horizontal gene transfer [3,8]. Moreover, as microbes become resistant to a specific drug, the subsequent use of alternative antibiotics might select for additional resistances, thus leading to the increasing menace of multidrug-resistant strains [2,9–11]. This is a prevalent scenario in *Staphylococcus aureus*, *E*. *coli*, or *Mycobacterium tuberculosis*, for instance, in which multiple resistances pose a serious threat to human health [9,12–14]. Thus, disclosing the evolutionary factors governing the maintenance of multiple resistance is key for designing effective treatments.

Chromosomal mutations conferring resistance, the focus of this study, are typically deleterious in the absence of antibiotics [15,16], since often they affect proteins involved in essential cellular machinery, such as the ribosome (i.e., streptomycin) [17] or the RNA polymerase—RNAP—(i.e., rifampicin) [18–20]. Nevertheless, bacteria can curb this deleterious effect by acquiring compensatory mutations [15,21–23]. This allows antibiotic resistance to become stabilized in the population while paying little or even no cost [24]. Acquiring additional mutations to overcome the fitness cost of resistance is also more likely to occur than the genetic reversion of the resistance mutation, since the range of targets for compensation is much broader [24,25]. Compensation for the cost of resistance has been widely described both in clinical [11,19] and laboratory [17,18,20] settings. The dynamics of compensatory adaptation depends on population parameters, such as bottlenecks [17] and mutation rate [21], and on the distribution of fitness effects of compensatory mutations, which depends on the genetic background [22].

Studies of compensation have focused on single resistances (e.g., [17,20,23,26]). However, resistance mutations often interact such that the fitness cost of multiple resistance tends to differ from that expected given the effects of each resistance [24,27]. These epistatic interactions might originate from the fact that resistance mutations affect interconnected functions in the cell [2]. Thus, cooccurring resistance mutations can lead to synergistic or antagonistic functional interactions [24–26]. This leads us to hypothesize that compensatory evolution of multiple-resistant bacteria may substantially differ from that of single-resistant strains. This may be especially important for chromosomal resistance mutations that exhibit negative epistasis, since the proteins involved in the interactions between the resistance mutations (which potentially contribute to the epistasis) could become targets for compensation. In that scenario, additional mutations beyond those that compensate for each individual resistance may be expected to occur. Thus, we further postulate that mutations that specifically compensate for epistasis should emerge during the evolution of multiple-resistant bacteria. To test these hypotheses, we followed the compensatory adaptation of single- and double-*E*. *coli*–resistant strains in antibiotic-free media. We chose three founder genotypes carrying chromosomal resistance mutations as a case study: RpoB^H526Y^ (conferring resistance to rifampicin), RpsL^K43T^ (conferring resistance to streptomycin), and a strain harboring the two mutations (RpoB^H526Y^ RpsL^K43T^), which show negative epistasis, causing a strong decrease in bacterial fitness [24,28].

To dissect the process of compensation for the mentioned founder genotypes, we analyzed the dynamics of the frequencies of neutral markers during compensatory evolution, performed whole-genome sequencing of evolved clones and allelic reconstruction of putative compensatory mutations in all genetic backgrounds. Our results demonstrate faster compensation in low-fitness double-resistant bacteria and unveil the identity of mutations that compensate for the negative epistasis between resistances. These mutations are advantageous only for double-resistant genotypes, i.e., they are either neutral or deleterious for single resistant and sensitive clones, as well as in environments where the epistasis between resistances is absent.

## Results

### Double-resistant bacteria compensate faster than single-resistant strains

**Fig 1:**
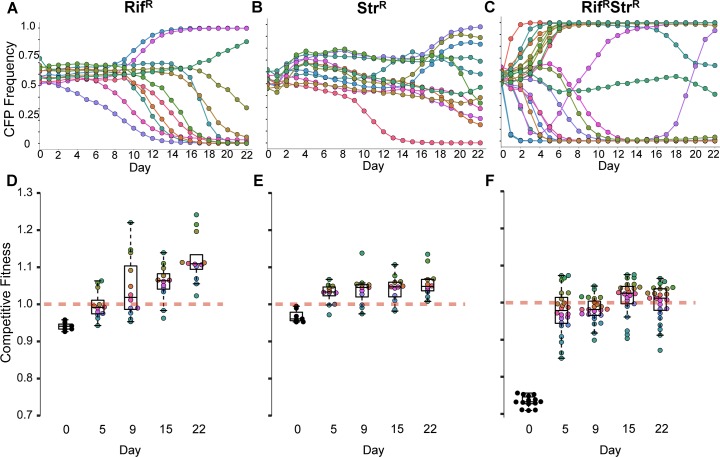
Faster compensatory evolution in double-antibiotic–resistant bacteria. (**A-C)** Dynamics of a fluorescent neutral marker during compensation in rich media without antibiotics of ~1:1 mixtures of yellow fluorescent protein (YFP)/cyan fluorescent protein (CFP) cells in (A) 12 independent *E*. *coli* populations resistant to rifampicin (RpoB^H526Y^); in (B) 12 populations resistant to streptomycin (RpsL^K43T^), and in (C) 24 populations resistant to both rifampicin and streptomycin (RpoB^H526Y^ RpsL^K43T^). **(D-F)** Competitive fitness of RpoB^H526Y^ (D), RpsL^K43T^ (E), and RpoB^H526Y^ RpsL^K43T^ (F) evolving populations, at different days during adaptation, measured against a reference nonfluorescent-sensitive strain. Each circle corresponds to a population with similar colour shown in panels A, B, and C, respectively. Red dashed lines correspond to the competitive fitness of the sensitive strain. The black dots at time 0 represent the competitive fitness of each of the founder resistant genotypes. Box plots represent the median and quartiles Q2 and Q3, and whiskers show the last quartiles of the data.

To study the dynamics of compensation and determine its pace, we performed experimental evolution in an antibiotic-free medium of six *E*. *coli* clones representing three founder genotypes, each carrying either of two neutral markers (Cyan fluorescent protein [CFP] or yellow fluorescent protein [YFP], **S1 Fig**): two carrying a Rif^R^ (RpoB^H526Y^) allele, two carrying a Str^R^ (RpsL^K43T^) allele, and two carrying both resistant alleles Rif^R^ Str^R^ (RpoB^H526Y^ RpsL^K43T^). The single resistances cause different fitness costs (0.06 ± 0.001 for RpoB^H526Y^ and 0.03 ± 0.01 for RpsL^K43T^) and generate strong negative epistasis—0.27 ± 0.01 >> (0.06 ± 0.01) + (0.03 ± 0.01)—in the double mutant (see **S2 Fig**). The neutral markers allow us to readily identify the emergence of compensatory mutations, as the frequency of a neutral marker is expected to rapidly increase when adaptive mutations sweep through evolving populations. Conversely, a marker should decrease in frequency when a beneficial mutation occurs in the subpopulation carrying the other marker. The number of generations, together with the relatively large population size and the mild bottlenecks during the propagations (see materials and methods), ensure that adaptation will mostly be driven by positive selection [29]. However, it is possible that some alleles that increase in frequency are neutral (or even slightly deleterious) and arise through genetic hitchhiking with beneficial mutations [30,31]. We followed the changes in marker frequencies over 22 days (~180 generations) in 12 independently evolving Rif^R^ populations, 12 Str^R^ populations, and 24 Rif^R^ Str^R^ populations (**Fig 1A–1C**).

We observed that, for the double-resistant Rif^R^ Str^R^ population (**Fig 1C)**, markers deviate from their initial frequency faster and with steeper slopes than for either of the single-resistant bacteria, Rif^R^ (**Fig 1A**, One-way ANOVA with Tukey’s Honest Significant Difference [HSD] correction, *p* < 0.0001) or Str^R^ (**Fig 1B**, One-way ANOVA with Tukey’s HSD correction, *p* = 0.001). In only 4 days of evolution, all double-resistant populations show signature of evolutionary adaptation, indicated by the changes in the frequency of the markers, that either reach frequencies close to fixation (>0.95) or show strong fluctuations. The latter indicate competition between clones carrying adaptive mutations (clonal interference) in both CFP and YFP backgrounds. Rapid and strong changes in marker frequency dynamics are expected for genetic backgrounds with low fitness—as it is the case for the double-resistant clones—which have been reported to adapt faster [32–35]. In single-resistant clones, near fixation of a marker is observed in a minority of the populations (three out of 12 Rif^R^ and 1 out of 12 Str^R^ populations) and only much later (8–10 days). Interestingly, the dynamics of Rif^R^ populations show no strong signs of clonal interference, whereas this phenomenon can be observed in a few Str^R^ populations. This suggests that more targets for adaptation may be available for Str^R^ than for Rif^R^, albeit with smaller effects.

### Faster compensation of double-resistant bacteria is driven by strong-effect mutations

To understand which process, mutation or selection, is responsible for the large differences in the dynamics of compensation of double **versus** single resistances, we measured the competitive fitness of the evolving populations along the adaptive process (defined by the increase in competitive ability of the evolving populations against a nonfluorescent sensitive strain, see materials and methods). A clear increase in competitive fitness is observed in all resistant backgrounds along the propagations (*t*-test *p* = 1x10^−6^ for Rif^R^, *p* = 6x10^−6^ for Str^R^, and *p* < 2x10^−16^ for Rif^R^ Str^R^, at day 22 of evolution, **Fig 1D–1F**), but the dynamics of fitness change differ among the different evolved resistant populations. The first 5 days show a strong and fast competitive fitness increase in Rif^R^ Str^R^ bacteria compared to the other backgrounds (**Fig 1D–1F**, ANCOVA, F(2,70) = 45.08, *p* < 0.0001, with 0.048 ± 0.003 fitness increase per day, between day 0 and day 5, significantly higher than Rif^R^ [0.01 ± 0.004] or Str^R^ [0.01 ± 0.004]) backgrounds. After day 5, the Rif^R^ populations have a more pronounced fitness increase along time compared to the other two backgrounds, in which competitive fitness seems to approach stabilization (generalized linear mixed effects model [GLMEM], Chi^2^{2} = 362.9, *p* < 0.0001, with 0.006 ± 0.0006 competitive fitness increase per day between day 5 and day 22, versus Str^R^ [0.0015 ± 0.0006] and Rif^R^ Str^R^ [0.0017 ± 0.0004]). Overall, the high initial cost of the double resistance (compare black dots with red dashed line in **Fig 1F**) is, on average, largely mitigated at day 5 (0.24 mean increase in competitive fitness, calculated as the difference between the competitive fitness of the evolved populations at day 5 and that of the founder double mutant, standard deviation [SD] = 0.06, across the 24 populations). Interestingly, the fitness increase of double-resistant bacteria appears to slow down at different fitness levels in the different populations. For instance, in population six, no significant fitness increase above 0.32 is observed after day 5 (linear regression’s differential fitness [dW] = y = −0.0005x + 0.32; R^2^ = 0.024), whereas for population 12, no significant gain above 0.15 is detected from day 9 onwards (dW = −0.001x + 0.149; R^2^ = 0.08, see **Fig 1F** and also **S3 Fig**). This suggests that the first step of adaptation may involve mutations with different fitness effects, which might condition the following adaptive steps.

**Fig 2:**
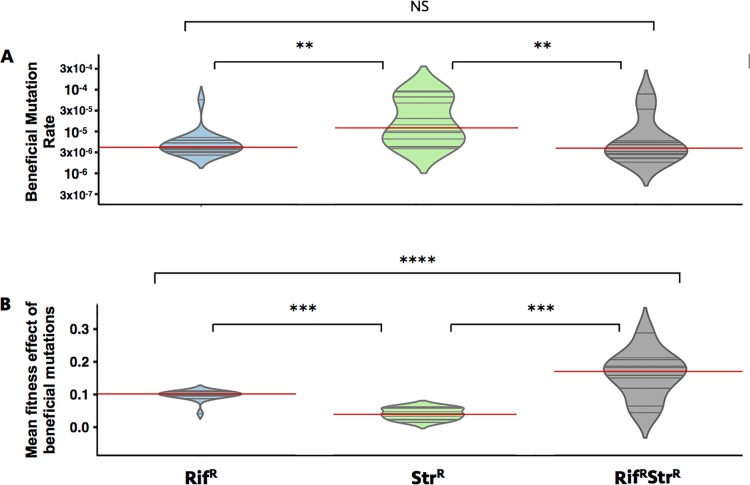
Compensatory mutations have stronger effects in double-resistant mutants. Estimates of the rate of acquisition of beneficial mutations (A) and mean selective effects (B) of arising beneficial mutations are shown in the violin plots and correspond to the distribution of the top 20 simulations, from the inference algorithm in the One Bi-Allelic Marker Algorithm (OBAMAv2), that best explain the experimental data. Red lines indicate the median of the distributions. NS, non-significant, **p < 0.01 and ****p < 0.0001 in a two-sample Kolmogorov test for comparing the distributions, adjusted for multiple comparisons using the Benjamini and Hochberg method [38].

The faster fitness increase in double-resistant bacteria can result from a higher rate of acquisition of beneficial mutations (due to, for instance, a larger target size) and/or from acquisition of mutations with stronger beneficial effects. To infer the rate and the distribution of effects of arising beneficial mutations in each of the backgrounds, we used a modified version of the One Bi-Allelic Marker Algorithm (OBAMAv2) [36] that considers both the marker dynamics and the fitness trajectories along the adaptive walk (see materials and methods). OBAMAv2 infers that the rate of acquisition of beneficial mutations (U) is not significantly different between the double resistant and the Rif^R^ populations (U ~ 3x10^−6^ per cell and generation), being higher in the Str^R^ background (U ~ 10^−5^ per cell and generation) (**Fig 2A**). This suggests a larger target size for compensation of Str^R^. However, the mean selective effect of beneficial mutations is higher for the Rif^R^ Str^R^ background than for either of the single-resistance backgrounds (0.18 for the double versus 0.1 for the Rif^R^ and 0.05 for the Str^R^)(**Fig 2B**). This could be due to the acquisition of similar mutations causing different effects across backgrounds with different initial fitnesses, as has been previously observed [33,34,37]. It may also be that the mutations underlying compensation could be different in single- and double-resistant bacteria. In the latter case, double-resistant bacteria would have access to mutations with higher mean selective benefit, which could potentially include compensatory alleles for the high cost resulting from the epistasis between Rif^R^ and Str^R^.

### Genetic basis of compensation in single- and double-resistant backgrounds

**Fig 3:**
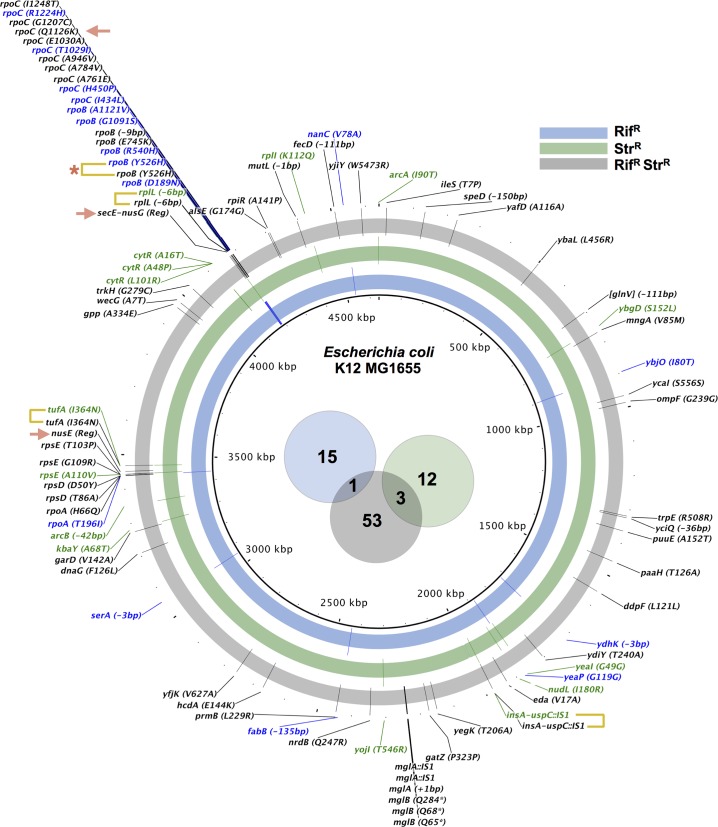
Genetic basis of compensation across resistant backgrounds. Mutations identified through population sequencing in each evolved antibiotic-resistant background. Mutations detected at least in one population map at the position indicated in the circular plot (see also **S1 Table**). The blue track and labels indicate mutations found in the 12 independently evolved Rif^R^ populations. The green track and labels indicate mutations found in the 12 independently evolved Str^R^ populations. The grey track and the black labels indicate mutations found in the 24 independently evolved Rif^R^ Str^R^. Mutations found in both a single- and the double-resistant backgrounds are indicated by yellow brackets (* indicates a back-mutation). Mutations in regulatory regions are indicated with (Reg). Mutations selected for allelic reconstruction and competition experiments are marked with an arrow. The Venn diagram shows the number of mutations specific to each background and in common between backgrounds (shown as blue, green, and grey).

To identify the compensatory mutations emerging in each of the studied founder genotypes, we performed whole-genome sequencing of pools of evolved clones. We pooled one clone from each evolved population carrying the fluorescence marker with the highest frequency at the end of the experiment (**Fig 1A–1C**). This allows capturing a broad sample of the compensatory landscape (**Fig 3**). We found mutations affecting compensatory targets previously described (10 out of 16 allelic changes in Rif^R^, 2 out of 15 in Str^R^, and 16 out of 57 in Rif^R^ Str^R^), such as *rpoB* itself, *rpoA*, and *rpoC* for rifampicin resistance and *rpsE*, *rpsD*, and *tufA* for streptomycin resistance (**Fig 3**). Indeed, several mutations found, affecting ribosomal genes, have been previously described as compensatory for the streptomycin resistance allele RpsL^K43N^ in *Salmonella* Typhimurium [17] (RpsE^A110V^ in the Str^R^ background and RpsD^D50Y^ in the Rif^R^ Str^R^ background) or affect similar residues (RpsD^T86A^, RpsE^T103P^, and RpsE^G109R^ in the Rif^R^ Str^R^ background). In addition, mutations in *rpoA* and in *rpoC* found in the Rif^R^ background (RpoA^T196I^ and RpoC^H450P^, respectively) were also previously identified as compensatory for the allele RpoB^H526Y^ [22]. We identified some putative compensatory targets that are common to single- and double-resistant backgrounds. At the level of functional category, we observed overlap in genes encoding membrane proteins (i.e., *ybjO* and *nanC* in Rif^R^; *cdgI* and *yojI* in Str^R^; *ompF* and *ydiY* in Rif^R^ Str^R^) and ribosomal proteins (*rplL* in Rif^R^ Str^R^ and *rplI* in Str^R^). At the gene level, there is a high parallelism, mostly involving the known compensatory targets previously mentioned, such as *rpoA*, *rpoB*, *rpoC*, *rpsE*, and *tufA*. At the nucleotide level, we found three mutations common to the Str^R^ and the double mutant: an insertion sequence [IS] element in a known hotspot for IS elements upstream from the *flhDC* operon [39], a nonsynonymous change in *tufA*, and a 6 bp deletion in *rplL*. A single compensatory mutation was found in common between Rif^R^ and the Rif^R^ Str^R^ backgrounds: a reversion in *rpoB* (**Fig 3** and **S1 Table**). Reversions are typically rare [21,40], but clinically relevant, since bacteria regain sensitivity to the antibiotic. To determine the extent to which reversion to sensitivity occurred in our evolving populations, we phenotyped clones (*n* > 40) from each evolved population at day 22. We streaked each clone sequentially on both the medium supplemented with antibiotic(s), at a concentration similar to where the founder genotypes were originally selected (i.e., 100 ug/ml of rifampicin, streptomycin, or both drugs), and in the antibiotic-free medium. We define “sensitivity” as the inability of a clone to grow in the presence of the antibiotic but able to grow in the drug-free medium. Remarkably, we found that, in 6 out of 24 populations of the Rif^R^ Str^R^ background, sensitivity to either rifampicin or streptomycin emerged. We also found the emergence of sensitive clones in the single-resistance evolved populations: in the Rif^R^ background, in two populations, fixation of sensitive clones was detected; in the Str^R^ background, the frequency of streptomycin-sensitive clones reached 64% (58%–71%, 95% confidence interval [CI]) in one population and 69% (62%–75%) in another (see **S2 Table**). Curiously, while the restoration of rifampicin sensitivity is mediated by a genetic reversion (RpoB^Y526H^), the tested clones that regained sensitivity to streptomycin still harbor the original mutation (RpsL^K43T^) and thus experienced a phenotypic reversion. Notably, phenotypic reversion of streptomycin resistance due to mutations in *tufA* (which appeared as target in our study) has been previously reported [41,42].

Genetic reversions are expected to cause strong beneficial effects in the double-resistant background, given that they provide a direct solution for the negative epistasis. For instance, a reversion in *rpoB* in the double-resistant bacteria would cause a 24% fitness increase, from a competitive fitness of 0.73 (27% cost) to 0.97 (3% cost) (see **S2 Fig**). Its rarity in the Rif^R^ Str^R^ background is a further indication of the emergence of other compensatory mutations with strong fitness effects, which is consistent with the very large fitness gains observed in the double-resistant populations (**Fig 1C** and **Fig 1F**). Interestingly, mutations affecting genes involved in DNA replication and DNA repair (i.e., *dnaG* and *mutL*) were detected in double-resistant evolved populations (**Fig 3**). Such mutations may have caused mutation rate changes in some of the evolved clones, thus resulting in a higher number of mutations accumulated. However, since the frequency of these mutations in the pooled run was only 3.6%, these mutations appeared in only a few populations (two at most, in the case of *mutL*) and thus are unlikely to have affected the estimation of evolutionary parameters (**Fig 2**). Apart from the previously mentioned IS element detected in some populations, both in the Str^R^ and Rif^R^ Str^R^ populations (**Fig 3**), no other types of structural variation, such as duplications, could be found, as assayed by looking for increases in coverage for any given region of the sequenced evolved lines.

In summary, the limited overlap among nucleotide changes across backgrounds suggests that single- and double-resistant bacteria acquire distinct compensatory mutations. This might be due, at least partially, to the existence of mutations that compensate for the epistasis between resistances in double-resistant bacteria.

### Compensation for the epistasis between resistances

**Fig 4:**
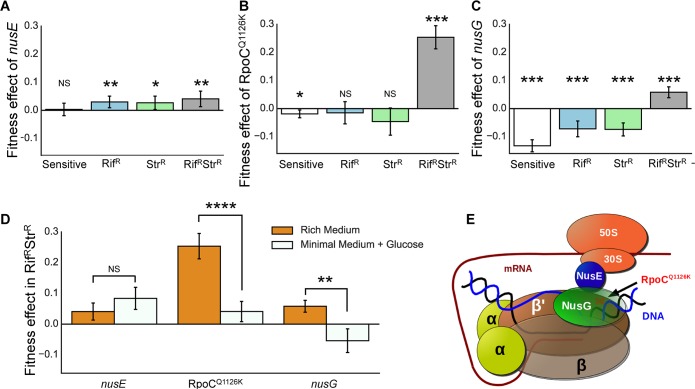
Mutations specifically compensating for epistasis between Rif^R^ and Str^R^. Fitness effect of specific compensatory mutations across backgrounds. Strains were constructed with each compensatory allele and competed against their corresponding ancestral (i.e., the resistant or the sensitive strain without the compensatory mutation). Mean values shown in columns (assays based on four independently constructed clones for each allelic replacement, each with at least *n* = 3 competitions, * *p* < 0.05, **p < 0.01, ***p < 0.001 from a one sample *t*-test), error bars indicate twice the Standard Error of the Mean (2SEM). (A) *nusE* is beneficial in all resistance backgrounds and neutral in the sensitive background (s = 0.04 ± 0.03 [2SEM], *p* = 0.01 for Rif^R^ Str^R^ background; s = 0.03 ± 0.02 [2SEM], *p* = 0.037 for Str^R^ background; s = 0.03 ± 0.02 [2SEM], *p* = 0.008 for Rif^R^ background; s = 0.003 ± 0.002 [2SEM], *p* = 0.774 for sensitive background, one-sample *t*-test). (B) RpoC^Q1126K^ shows a strong benefit in the double-resistance background (0.25 ± 0.04 [2SEM], *p* < 0.0001), whilst being neutral in Rif^R^ (s = −0.02 ± 0.04 [2SEM], *p* = 0.457) and deleterious in both the Str^R^ and the sensitive backgrounds (s = −0.05 ± 0.05 [2SEM], *p* = 0.085 or (s = −0.02 ± 0.01 [2SEM], *p* = 0.015) for the RpsL^K43T^ or the sensitive, respectively). (C) *nusG* is beneficial in the double-resistance background (s = 0.06 ± 0.02 [2SEM], *p* < 0.0001) and deleterious in all other backgrounds (s = −0.07 ± 0.02 [2SEM], *p* < 0.0001 for Str^R^ background; s = −0.07 ± 0.03 [2SEM], *p* < 0.0001 for Rif^R^ background; and s = −0.132 ± 0.02 [2SEM], *p* < 0.0001 for sensitive background). (D) The benefit of RpoC^Q1126K^ mutation is significantly reduced and *nusG* is deleterious in minimal media supplemented with glucose (M9+Glucose), where the epistasis between Rif^R^ and Str^R^ does not take place. However, *nusE*, which does not seem to specifically compensate for the epistasis, is beneficial regardless of the media. (E) Schematic representation of the coupling between the RNA polymerase (RNAP) and the ribosome mediated by the NusE–NusG complex. A red cross represents the approximate position of RpoC^Q1126K^ in the domain of RpoB that NusG binds to.

To test the hypothesis that epistatic interactions between the resistance alleles provide additional compensatory targets, we generated derivatives of all resistant backgrounds harboring selected compensatory mutations that we have only found in double-resistance populations and could potentially compensate for the epistasis. We used genetic reconstruction to isolate the compensatory effect of each individual mutation for double-resistant bacteria, as well as measuring its fitness effect in the sensitive and single-resistant backgrounds. To do so, we performed competitions between derivatives of the founder genotypes (RpoB^H526Y^, RpsL^K43T^, or RpoB^H526Y^ RpsL^K43T^) harboring the compensatory mutation(s) and their corresponding ancestral (not carrying the compensatory mutation) and estimated their fitness effect from differences in growth over a 24 h competition (see materials and methods). We selected a single nucleotide polymorphism (SNP) in the *rpoC* gene (RpoC^Q1126K^) because, although *rpoC* is a common compensatory target for rifampicin resistance [19,26,43], this change was detected at a very high frequency (26.1%) in the double Rif^R^ Str^R^ populations but not detected in any of the single-resistant populations (**Fig 3** and **S1 Table**). Additionally, we chose two regulatory mutations observed exclusively in the double-mutant background: one in the promoter region of the *secE-nusG* bicistronic transcript [44] (*nusG* hereafter), and another in a regulatory secondary structure in the 5′ untranslated region (5′-UTR) of the operon that encodes *rpsJ* (*nusE*) [45] (*nusE* hereafter). The latter two mutations were selected because they are good candidates for being compensatory to epistasis, since NusG and NusE mediate the molecular interaction between the ribosome and the RNAP [46] (**Fig 4E**).

We found the *nusE* mutation to be beneficial across all resistance backgrounds but neutral in the sensitive bacteria (**Fig 4A** and **S4A Fig**). In contrast, the mutation in *rpoC* confers a strong beneficial effect only in the double Rif^R^ Str^R^ background, where it emerged, being neutral or even slightly deleterious in either the single-resistance or the sensitive backgrounds (**Fig 4B** and **S4B Fig**). Finally, the *nusG* mutation is exclusively beneficial in the double Rif^R^ Str^R^ background, being highly deleterious in all other backgrounds (**Fig 4C** and **S4C Fig**). Therefore, the beneficial effects of RpoC^Q1126K^ and *nusG* compensatory mutations are strongly conditioned by the presence of the two resistance alleles, suggesting that, in both cases, the target of compensation is the epistatic interaction between the resistance alleles. To further corroborate this we measured the effects of RpoC^Q1126K^ and *nusG* mutations in minimal medium supplemented with glucose, an environment where RpoB^H526Y^ and RpsL^K43T^ were previously shown not to exhibit negative epistasis [28]. We found that RpoC^Q1126K^ loses most of its beneficial effect in this environment (from 25.4% to 4%), indicating that it partially compensates for the epistasis. Remarkably, *nusG* is indeed deleterious in minimal medium supplemented with glucose, demonstrating that this mutation compensates specifically for the epistasis between antibiotic resistances (**Fig 4D**, and **S4B** and **S4C Fig**). On the other hand, *nusE* does not seem to compensate specifically for the negative epistasis (**Fig 4A**), as it is equally beneficial in minimal medium supplemented with glucose, where the epistasis is not present. In sum, we found that while *nusE* is a global compensatory mutation to the costs of resistance, RpoC^Q1126K^ and *nusG* are highly idiosyncratic and only likely to be found linked to double-resistant backgrounds, since they compensate for the negative epistasis between these resistance alleles.

**Fig 5:**
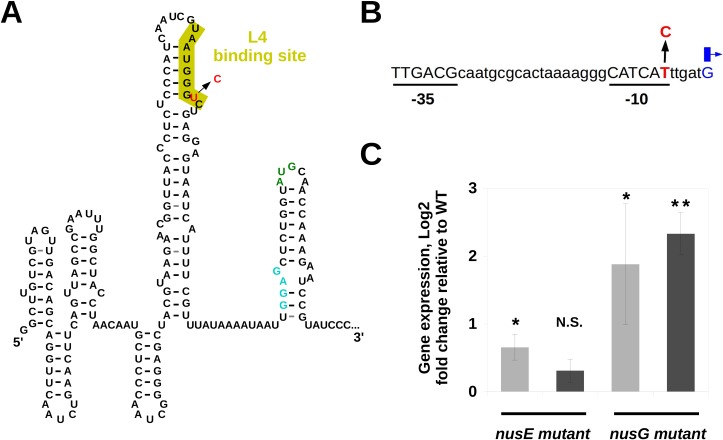
Location and effects of the mutations in the regulatory regions of *nusE* and *nusG*. (**A**) Structure of the leader transcript of the *nusE* operon as determined in [50]. The ribosome binding site and the translation start codon are shown in light blue and green, respectively. The mutated nucleotide (red) is located in a region (marked in yellow) essential for the attenuation mediated by L4 [45,47,51]. (**B**) Sequence of the promoter region of the *secE-nusG* transcript. The transcription start site is indicated by the blue arrow. The mutation (red) affects the −10 sequence [44]. (**C**) Log2 fold change of the expression of *nusE* (light grey) and *nusG* (dark grey) genes in each mutant with respect to the wild type, measured by quantitative real-time PCR (RT-qPCR) (from left to right, mean ± twice the standard error of the mean [2SEM], *p*-values of one sample *t*-test are: 0.65 ± 0.19, *p* = 0.020; 0.31 ± 0.17, *p* = 0.068; 1.88 ± 0.89, *p* = 0.052; 2.33 ± 0.31, *p* = 0.004; *n* = 3).

The striking difference in the fitness effects of *nusE* and *nusG* regulatory mutations prompted us to analyze their effects on gene expression in the sensitive background by quantitative real-time PCR (RT-qPCR). The change in the leader transcript of *nusE* (**Fig 5A**) caused a small but significant increase (less than 2-fold) in its expression level (**Fig 5C**), consistent with the mutation affecting a region involved in its transcriptional and translational attenuation [47]. The mutation in *nusG* affects the −10 sequence of its promoter region (**Fig 5B**) [44], resulting in a strong increase (above 4-fold) in its expression (**Fig 5C**, rightmost column). Interestingly we found that the *nusG* mutation also causes upregulation of *nusE* expression (**Fig 5C**, third column). This cross-regulation might be due to the fact that NusA participates in the attenuation of *nusE* operon [47] and that NusG can interact with NusA [48], interfering with its activity [49]. Importantly, the effects of the *nusE* and *nusG* regulatory mutations on gene expression reflect their effects on the fitness of sensitive bacteria: the strong and pleiotropic induction caused by the *nusG* mutation generates a high fitness cost (**Fig 4C**), while the milder and less pleiotropic change on expression caused by the *nusE* mutation entails a close to neutral fitness effect (**Fig 4A**). This suggests that the expression of these genes constitutes an important mechanism connecting genotype, phenotype, and fitness.

## Discussion

As spread of multidrug-resistant bacteria increases, it is crucial to understand how multiple resistant alleles can be maintained in populations [52]. Thus, investigating the process of acquisition of compensatory mutations is essential. Although compensation for single-resistance mutations has been thoroughly studied, our findings suggest that previous observations are not readily transferrable to compensation in multiresistant strains. We demonstrate that the pace of compensation is faster in a bacterium resistant to both rifampicin and streptomycin, due to stronger effect mutations being accessible to this low-fit background. Compensation by stronger effect mutations is supported by direct competitive fitness assays, in which high-fitness increments were detected (**Fig 1**). In particular, we found a mutation (RpoC^H450P^) beneficial in the Rif^R^ background (s = 0.05 ± 0.01) that shows a stronger beneficial effect (s = 0.10 ± 0.01) in the double mutant (**S5 Fig**). This is consistent with the general pattern recurrently observed that low-fitness genotypes tend to acquire stronger effect mutations [33,34,53,54] and tend to adapt at a faster pace [32,35]. Interestingly, when studying the evolvability of other Rif^R^ mutants, Barrick et al. found that lower-fitness genotypes were able to adapt faster due to access to stronger effect mutations [37]. Thus, our observations fit the previous found patterns.

Curiously, we observed that, in some adapted populations, the competitive fitness exceeds the initial fitness cost of the ancestral genotype (see **Fig 1D**). This indicates that either there is further adaptation to the environment (i.e., the ancestral genotype is not at or very near to the fitness peak) beyond compensation and/or that the initial resistance mutation displaced the genotype in the fitness landscape, allowing the exploration of other, potentially higher, fitness peaks [55]. In order to determine the extent to which adaptation to the environment occurs in our experimental conditions, we propagated sensitive bacteria during 22 days in an antibiotic-free medium, performed competitive fitness assays, and determined the mutation targets associated with such propagation (**S6 Fig**). We found that marker frequency changes at a much slower pace (**S6A Fig**), but it is not static, and associated increases in competitive fitness can be detected (**S6B Fig**). Therefore, other mutations associated with the adaptation to the environment occur. Thus, adaptation to the environment could also have occurred in the resistant backgrounds. We note, however, that we could not detect any common mutational targets between sensitive and resistant backgrounds (**S6C Fig**).

Interestingly, in the double-resistant backgrounds, the dynamics of fitness appear to stabilize at different plateaus for different populations (**Fig 1F** and **S3 Fig**). However, the period of propagation done in this study is too short to assess if these populations are indeed stabilizing at different peaks. Nevertheless, it is possible to conceive the existence of a complex network of epistatic interactions among the compensatory mutations. In that scenario, the initial acquisition of a given compensatory mutation could influence the fitness effects of subsequently acquired compensatory mutations, restricting the range of evolutionary trajectories during further adaptation [56].

When analyzing the genetic basis of compensation, we detected several new putative compensatory targets (**Fig 3**). In principle, the general lack of overlap across the three mutant backgrounds and the sensitive strain (**Fig 3**, **S6C Fig**, **S1 Table**) suggests that the majority of the mutations found are not due to general adaptation to the selected growth conditions but, instead, are driven by compensation to the cost of resistance. Moreover, although the target size for adaptation to growth conditions might be large enough to make overlap relatively infrequent, and the sampling method used (one clone per population) may limit detecting overlapping mutations, many of the targets identified are directly related to transcription and translation, suggesting that they are indeed compensatory.

The observation of parallelism in targets such as the *mglBAC* operon in the Rif^R^ Str^R^ populations or the *cytR* gene in Str^R^ populations suggests that targets of compensation not directly related to *rpsL* and *rpoB* might be available. By genetic reconstruction, we found that a *mglA* mutation is beneficial in both the Rif^R^ and Rif^R^ Str^R^ backgrounds, being deleterious in the Str^R^ background and neutral in the sensitive (**S7 Fig**). This points to the *mglA* mutation being indeed compensatory for Rif^R^ and not generally adaptive, which is also supported by its absence in the mutational targets detected from the propagation of the sensitive strain (**S6C Fig**). The functional unrelatedness of the *mglBAC* operon (sugar transport [57]) with the cellular machinery affected by the original Rif^R^ mutation (RNAP) suggests that the mutations in the *mglBAC* operon do not correct for the damage generated by the resistance mutation. Instead, they may somehow alleviate its consequence and, therefore, its fitness cost.

Several alleles reconstructed (RpoC^Q1126K^, *nusG*, and *nusE*) in the different backgrounds were found to be beneficial in the single- or double-resistant genotypes but not in sensitive bacteria, providing direct evidence of their compensatory role. The presence of these compensatory mutations has no discernible impact on the ability of sensitive, single-, or double-resistant bacteria to withstand rifampicin and streptomycin, as no significant change in their minimum inhibitory concentrations was detected (**S3 Table**).

The dynamics of marker frequency and the distribution of fitness effects in the propagation experiment (**Fig 1C** and **1F**) suggest that double-resistant bacteria already acquired compensatory mutations with large fitness effect by day 5. Indeed, population-sequencing of the evolved double-mutant bacteria at day 5 (**S1 Table**) confirmed that several mutations present at day 22 appear already at day 5 (for instance, RpsE^T103P^, RpoA^H66Q^, an IS insertion between *insA* and *uspC*, RpoB^E745K^, RpoC^A784V^, RpoC^Q1126K^, RpoC^E1030A^, and RpoC^I1248T^). The maintenance of these alleles in the populations suggests that their fitness effects are strong enough to have prevented them from being outcompeted by other mutants during the evolution experiment. Consistently, one of these mutations (RpoC^Q1126K^) was shown to cause a large compensatory effect (**Fig 4B**).

Among the compensatory mutations found, we identified specific sets of mutations with sign epistasis, i.e., that shift from beneficial to deleterious depending on the genetic background in which they occur (**Fig 4**). Indeed, we show that compensation in Rif^R^ Str^R^ bacteria can specifically target the epistatic interaction between the resistant alleles. Importantly, mechanisms specifically targeted by these compensatory mutations include the coupling between transcription and translation, modulating the interaction between the resistances themselves. For instance, NusG and NusE proteins, which constitute the molecular link between RNAP and ribosomes [46] (**Fig 4E**), are mutational targets of compensation in the double-resistant strains. NusE is part of the 30S ribosomal complex [58] and, besides connecting with the RNAP through NusG [46], it has been suggested to bind to it directly [59]. Therefore, a beneficial effect in the different resistance backgrounds, but not in sensitive bacteria, could be expected. Moreover, the regulatory mutation in *nusE* likely affects the expression of its entire operon, which encodes ten other ribosomal proteins, whose potential compensatory effects will have to be analyzed in future research. On the other hand, the mutation affecting NusG, a universally conserved transcription termination/antitermination factor, shows a beneficial effect specific to the epistasis between streptomycin and rifampicin resistance alleles. A tentative explanation for this effect relies on the coupling of transcription and translation in prokaryotes. RpoB^H526Y^ and RpsL^K43T^ resistance mutations generate dysfunctional RNAP [60] and ribosome [61], respectively, affecting transcription and translation efficiencies. These altered processivities likely generate discoordination of these two machineries, therefore challenging the transcription–translation coupling. In that scenario, mutations causing increased expression of factors involved in the physical interaction between the RNAP and the ribosome (NusG and NusE) [46] may help to enhance their coordination, restoring the coupling. Indeed, the RpoC^Q1126K^ mutation partially compensates for the epistasis between resistances, and the analysis of the *E*. *coli* RNAP structure available (PDB 3LUO) indicates that the residue Q1126 is at the surface of the RpoC protein and maps closely to the region of the protein described to interact with NusG (**Fig 4E** and **S8 Fig**). This suggests that the RpoC^Q1126K^ mutation could also be mechanistically linked to the interaction between the RNAP and the ribosome and, therefore, to transcription–translation coupling.

To the best of our knowledge, this is the first time that the NusG–NusE complex has been identified as a compensatory target for antibiotic resistance. This shows the power of experimental evolution to identify novel compensatory targets in resistant bacteria and also reveals its potential at uncovering important mechanisms in cellular fitness [62]. Moreover, the identification of mutations targeting the regulation of gene expression raises the possibility of the existence of nonmutational mechanisms for phenotypic compensation of the fitness cost of antibiotic resistances, as it has been recently described in mycobacteria [63]. Importantly, although our observations are limited to the set of resistance mutations studied, given that the mechanisms involved in their interaction (transcription and translation) are commonly targeted by antibiotics [64,65], these observations likely apply to other resistance mutations. It will be interesting to study other multiresistant backgrounds in the future in order to test this possibility.

Resistance mutations typically affect essential cellular functions and more often than not those mutations tend to exhibit epistasis [28,66]. Indeed, observations in clinical populations of bacterial pathogens suggest the existence of undiscovered epistatic interactions [67]. Thus, there is an urgent need to uncover the spectrum of mutations that suppress the costs arising from epistatic interactions in multidrug-resistant pathogens. Our results suggest that this cannot be achieved by focusing on the study of single-resistant strains. The ability to identify and determine how specific mutations affect epistasis between resistant alleles is essential for understanding how antibiotic resistances persist in bacterial populations. Interestingly, this knowledge may provide new grounds for the development of novel antimicrobial strategies that specifically exploit potential weaknesses derived from epistasis between antibiotic resistances in multidrug-resistant bacteria [68].

## Materials and methods

### Strains and growth conditions

All the strains used in this study (**S4 Table**) are derivatives of an *E*. *coli* K12 MG1655 marked with constitutively expressed YFPs or CFPs inserted in the *galK* locus and the entire *lac* operon deleted (*ΔlacIZYA galK*::*cat*::*PLlacO-1-YFP/CFP*). Resistant mutants also harbor chromosomal-resistance mutations to either streptomycin (RpsL^K43T^ allele), rifampicin (RpoB^H526Y^ allele), or both. Single- and double-resistance strains in both fluorescent backgrounds were used in the long-term propagation experiment in Lysogen Broth (LB) without antibiotics. Cultures were grown in a 96-well plate incubator at 37°C with shaking (700 rpm). Nonfluorescent wild-type *E*. *coli* K12 MG1655 was used as reference strain for the competition fitness assays, except when specified otherwise.

### Experimental evolution for compensation

In order to acclimatize bacteria to the environment, strains were grown separately from frozen stocks in LB media (150 ul per well) in 96-well plates at 37°C with shaking (12 replicates per strain were inoculated in a checkered format to avoid cross-contaminations). After 24 h, 10 ul of bacteria culture diluted by a factor of 10^−2^ was transferred into 140 ul fresh LB medium and allowed to grow for an additional 24 h. Isogenic strains differing only in the marker were diluted again by a factor of 10^−2^ and then mixed based on their cell numbers given by the Flow Cytometer (LSR Fortessa) in order to obtain an initial ratio of 1:1. CFP was excited with a 442 nm laser and measured with a 470/20 nm pass filter. YFP was excited using a 488 nm laser and measured using a 530/30 nm pass filter. A total of 48 competitions were initiated by inoculating 140 ul of LB medium with 10 ul of each mixed population, which were allowed to grow for 24 h, reaching a concentration of, approximately, 10^9^ CFU/ml. The separate growths were done to minimize the occurrence of common compensatory mutations during acclimatization, a phenomenon that is difficult to avoid. After every 24 h of growth, and for 22 days, these cultures were propagated by serial passage with a constant dilution factor of 10^−2^ (10 ul of diluted culture was transferred into 140 ul of fresh medium). In parallel, cell numbers were counted using the Flow Cytometer in order to measure the frequency of each strain in the mixed population during the experiment, by collecting a sample (10 ul) from the spent culture each day. Samples were frozen at days 5, 9, 12, 15, 18, and 22.

### Competitive fitness assays

The relative fitness of each evolved population at the end of the propagation experiment, at day 22, was measured by competitive growth against the nonfluorescent (unmarked) isogenic reference strain *E*. *coli* K12 MG1655. The competitor (each evolved population, potentially composed of both YFP and CFP) and the reference (unmarked) strains were first unfrozen and acclimatized separately for 48 h (with two growth periods of 24 h) and then mixed in a proportion of 1:1, using a method similar to the one previously described. To assess the cost of the resistances themselves before any compensation, control competitions were performed between the ancestral of each resistant mutant and the reference strain by mixing 25% of YFP + 25% of CFP with 50% of the unmarked strain. Thus, a total of 52 competitions were initiated by inoculating 140 ul of LB media with 10 ul of each mixed population and allowed to compete for 48 h. The initial and final frequency of the strains were obtained by counting their cell numbers in the Flow Cytometer. Generation time was estimated from the doubling time of the reference strain (approximately eight generations), and the fitness was determined as the average of three independent replicates for each competition.

### Sensitivity assay of evolved populations

Evolved cultures were grown in 96-well plates with LB for 24 h and subsequently plated in antibiotic-free solid media. From each evolved population, clones were picked with a pipette tip, which then was used to streak sequentially both on antibiotic plates (containing 100 ug/ml of rifampicin, streptomycin, or both drugs) and antibiotic-free plates (LB agar only). Clones were classified as sensitive if they grew on an antibiotic-free media but not on media with antibiotic(s). Fifty clones were initially tested for each population. Populations in which sensitive clones were found were subsequently tested for another 150 clones.

### Estimation of evolutionary parameters

The method used to estimate the rate of acquisition of beneficial mutations and the distribution of selective effects was modified from the one published in [36]. Briefly, the method simulates 1 million marker dynamics with parameters (mutation rate, shape, and mean of a gamma distribution for the selective effects of new mutations) chosen from a uniform distribution. Afterwards, marker frequency and average population fitness are used to summarize both the experimental data and the simulated dynamics. These data are used as summary statistics to be compared by Approximate Bayesian Computation (ABC). The modification in the method done here is that a simple ranking and rejection method was used (instead of using the neural network function in ABC), in which simulations are ranked by the Euclidean distance between summary statistics from the simulated and experimental data, and the top 20 simulations are chosen for the distributions of parameters. This change in the method was required, as the neural network method failed to provide biological reasonable parameters for the observed experimental data. We note that, in our estimates, we assumed that mutations can only arise during the simulated period (t > 0). Thus, we have ignored the short period of acclimatization. Given the possibility that mutations might have occurred during acclimatization, this could cause an overestimation of the rate of appearance of beneficial mutations.

### DNA extractions

The evolved populations were plated onto LB agar plates and grown for 24 h at 37°C. One colony, with the most frequent marker, was picked from each population and was grown overnight into 10 mL of liquid LB, at 37°C with shaking. The bacterial DNA was then extracted, and its concentration and purity were quantified using Qubit and NanoDrop, respectively. Following quantification, equal concentration of each DNA sample was taken and pooled together with the clones from the populations of a similar resistant background, resulting in three pools of DNA (Rif^R^ pool with 12 clones, Str^R^ pool with 12 clones, and Rif^R^ Str^R^ pool with 24 clones).

### Whole-genome sequencing analysis

The DNA extracted was sequenced using the Miseq Illumina platform. Coverage of the different pools was as follows: 521x for 12 clones in the Rif^R^ pool, 640x for 12 clones in the Str^R^ pool, and 631x for 24 clones in the Rif^R^ Str^R^ pool. The reads were filtered using SeqTk version 1.0-r63. The resulting sequences were analyzed in Breseq version 2.3, using *E*. *coli* K12 genome NC_000913.2 as a reference, and with the polymorphism option selected and the following parameters: (a) rejection of polymorphisms in homopolymers of a length greater than three, (b) rejection of polymorphisms that are not present in at least three reads in each strand, and (c) rejection of polymorphisms that do not have a *p*-value for quality greater than 0.05. All other Breseq parameters were used as default. Mutations in homopolymers and pseudogenes were also discarded. The genomic data in **Fig 3** was plotted using the BLAST Ring Image Generator (BRIG) software [69].

### Allelic reconstructions and competitions

The mutations located in *nusE* and *nusG* regulatory regions were constructed by Lambda-Red recombineering [70] using a resistance cassette inserted in a nearby gene (*gspB* and *tufB*, respectively) as a marker for selection, followed by transference to Rif^R^, Str^R^, and Rif^R^ Str^R^ backgrounds by P1 transduction [71] using the same marker. Derivatives of these strains harboring the marker in the nearby gene but not the mutations in *nusE* and *nusG* were constructed, in order to be used as references in the competition experiments. The putative compensatory mutations located in *rpoC* were constructed by pORTMAGE recombineering [72] in both the Rif^R^ and the Str^R^ backgrounds and subsequent transfer to the sensitive, Rif^R^, Str^R^, and Rif^R^ Str^R^ backgrounds was done by P1 transduction, using as recipient auxotroph derivatives carrying a mutation in the nearby gene *argE* and selecting for growth in minimal medium. The primers used are listed in the **S5 Table**. The presence of the desired mutations in the transductant isolates was assessed by PCR-mediated amplification of the corresponding gene and sequencing. Both reconstructed strains and the corresponding reference strains were unfrozen and acclimatized during 24 h to avoid compensatory mutations appearing in the more costly backgrounds. Reconstructed mutants were competed against the respective ancestral strain to assess their competitive advantage per generation, assuming the competition lasts eight generations, or 24 h. The fitness landscapes of the main reconstructed compensatory genotypes (**S4 Fig**) were plotted using the MAGELLAN (Maps of Genetical Landscapes) software [73].

### RNA extraction, reverse transcription, and RT-qPCR

To determine changes in gene expression caused by the mutations in the regulatory regions of *nusE* and *nusG*, RNA extraction from 0.5 ml of cultures at the late exponential phase (OD_600_ 0.6) of isogenic strains harboring, or not, each mutation was performed using a Direct-Zol RNA miniprep kit (Zymo Research) according to manufacturer's specifications. Bacterial RNA was subsequently treated with RQ1 DNase (Promega) according to manufacturer's protocol. Reverse transcriptase reaction was performed on 880 ng of RNA, using M-MLV RT (Promega) and random primers (Promega) according to manufacturer's protocol. RT-qPCR was executed in a Bio-Rad CFX 384 Real-Time PCR Detection System, using iTaq Universal SYBR green Supermix (Bio-Rad). The cDNA was diluted 200-fold before being used for RT-qPCR. The cycling conditions were as follows: one step of 10 min at 95°C and then 40 cycles of 30 s at 95°C, 30 s at 67°C, and 30 s at 72°C. The primers used are listed in the **S5 Table**. Melting-curve analysis was performed to verify product homogeneity. All reactions included three biological replicates for each strain, and three technical replicates for each sample. For analysis, data were normalized using the algorithm specific for multiple reference genes described by Hellemans et al. [74], using *rrsA* and *ssrA* housekeeping genes as references.

### Minimal inhibitory concentration (MIC) determination

The MIC of rifampicin and streptomycin in the ancestral and the compensated backgrounds was determined by using MIC test strips (Liofilchem S.R.L.) in LB agar medium, ranging from 0.016 to 256 μg/ml for rifampicin and from 0.064 to 1,024 μg/ml for streptomycin. The strains resistant to the maximal concentrations in the MIC test strips were assayed for higher concentrations in liquid medium (LB broth), up to 768 μg/ml for rifampicin and 8,192 μg/ml for streptomycin.

### Statistical analysis

The assessment of different slopes for single and double mutants (**Fig 1A–1C**) was performed using a One-way ANOVA, corrected with Tukey’s HSD to compare between the different resistance backgrounds. The significance of the change in competitive fitness (**Fig 1D–1F**) was performed using an analysis of covariance (ANCOVA) between the ancestral populations and the evolved populations at day 5 and GLMEM (with restricted maximum likelihood) for the fitness changes between day 5 and the end of the experiment (day 22). In both cases, we have used Hochberg’s adjustment to correct for multiple testing. The differences in estimated rate of acquisition of beneficial mutations (**Fig 2A**), and also the differences in their effects (**Fig 2B**), were analyzed using a two-sample Kolmogorov, adjusted for multiple comparisons using the Benjamini and Hochberg method [38]. The significance of the fitness effect of compensatory mutations across backgrounds (**Fig 4A–4C** and **Fig 5C**) was assessed by ANOVA with Tukey's correction for multiple testing. The CIs for classifying a population “sensitive” (or partially sensitive) (**S2 Table**) were assessed using a Binomial proportion CI on the number of colonies assayed that show a sensitive phenotype.

## Supporting information

S1 Fig No cost of the fluorescence across backgrounds. Each fluorescent background (both CFP and YFP) was competed against the same genotype without the fluorescence. NS indicates non-significance according to a one sample T-test, to test deviation from neutrality (Sensitive, s = 0.038 (2SEM = 0.046), P = 0.158; Rif^R^, s = -0.002 (2SEM = 0.027), P = 0.867; Str^R^, s = -0.034 (2SEM = 0.035), P = 0.102; Rif^R^Str^R^, s = -0.052, (2SEM = 0.042), P = 0.066).
(PDF)

S2 Fig Competitive fitness of the resistant strains. The Rif^R^ Str^R^ background shows strong negative synergistic epistasis. Bars represent the mean fitness of the resistant strains when in competition with a sensitive, error bars represent 2SEM.
(PDF)

S3 Fig Dynamics of competitive fitness increase for the Rif^R^ Strep^R^ populations show stabilization at different levels of fitness. Error bars indicate the SEM across 3 independent replicates. In **A** are the dynamics of all the 24 populations, and in **B** are shown 6 indicative populations, for clarity.
(PDF)

S4 Fig Fitness landscapes of antibiotic resistant genotypes. Genotypes are characterized by the presence (closed dots) or absence (open dots) of three different alleles, representing, respectively, the RifR mutation, the StrR mutation and one compensatory mutation, which changes depending on the panel. Peaks, in green, indicate the maximum fitness, and sings, in red indicate the genotype with the lowest fitness of each landscape of 3 alleles. Closed dots in blue represent the genotype in minimal medium supplemented with glucose. In A) *nusE* (third allele) improves the position in the landscape for all genotypes, since it is beneficial across all backgrounds. In B) and C), RpoC^Q1226K^ and *nusG*, respectively, only improve the position of the double resistant genotype, with the magnitude of the improvement depending on the environment. Landscapes were constructed with the MAGELLAN software [73], and edited to include the genotypes in a different environments.
(PDF)

S5 Fig Higher compensatory effect of RpoC^H450P^ mutation in the double resistant background. Mean effect on fitness (± 2 SEM) of the mutation in the single (in blue) and double resistant (in grey) backgrounds.
(PDF)

S6 Fig Frequency marker dynamics and fitness increase in sensitive bacteria. (**A)** Dynamics of a fluorescent neutral marker during propagation in rich media without antibiotics of 1:1 mixtures of YFP/CFP cells in 12 independent sensitive *E*. *coli* populations. **(B)** Competitive fitness of the sensitive evolving populations, at different days during adaptation. Each circle corresponds to a population with similar colour shown in panels A. Red dashed lines correspond to the competitive fitness of the sensitive strain. Box plots represent the median and quartiles Q2 and Q3, and whiskers show the last quartiles of the data. **(C)** Genetic basis of adaptation of the sensitive bacteria. Mutations identified through population sequencing in the evolved antibiotic sensitive background. Mutations detected at least in one population map at the position indicated in the circular plot (see also S1 Table).
(PDF)

S7 Fig The *mglA* knockout mutation is beneficial in the in the Rif^R^ and the Rif^R^Str^R^ backgrounds. The knockout of *mglA* is beneficial in the Rif^R^ (s = 0.085 (2SEM = 0.035), P = 0.0006) and Rif^R^Str^R^ (s = 0.106 (2SEM = 0.031), P = 0.008) backgrounds, being neutral in the sensitive background (s = -0.014 (2SEM = 0.040), P = 0.483) and deleterious in the Str^R^ background (s = -0.07 (2SEM = 0.031), P = 0.001). Bars indicate the mean effect on fitness and error bars indicate twice the SEM.
(PDF)

S8 Fig Structure of the RNA polymerase complex, highlighting the location of the RpoC^Q1126K^ allele. Detail of RNA polymerase showing the β (in blue) and β’ (in brown) subunits encoded by the *rpoB* and the *rpoC* genes, respectively. The proposed binding area of NusG N-terminal domain [75–77] is highlighted with a black circle and it partially overlaps with the DNA binding domain (highlighted with an orange circle). The β’ clamp helices are crucial for the NusG interaction with RpoC.Residue RpoC^Q1126K^ (in red) is localized in a DNA binding region domain and maps closely to the proposed binding area of NusG. Figure drawn in Pymol using the crystal structure of *E*. *coli* RNAP (PDB 3LUO).
(PDF)

S1 Table Mutations acquired during the evolution in antibiotic-free media. Mutations are shown for the 3 different resistant backgrounds, along with the frequency at which they were detected. Mutations in known compensatory targets are marked in bold.
(XLSX)

S2 Table Sensitivity assays in the evolved populations. Column “Plated” indicates the total number of colonies (clones) that were inoculated on agar plates; The total number of those colonies that show reversion (i.e., sensitivity) to the antibiotic where they were being tested is indicated in column “# of Revertants in RIF/STRP/RIF+STREP”. The “Fraction Rev.” columns summarizes the total fraction of the inoculated clones (between 0 and 1) that became sensitive. Finally, the last columns, “Bin Err” shows the Binomial confidence interval for the sensitivity assay, according to the formula z*SQRT((1/n)*p(1-p)). (where **z** = 1-(1/2)*0.05, for a 95% confidence interval, **n** = number of trials and **p** = ns/n, with **ns** = number of successes). Populations were identified as sensitive (or at containing sensitive clones) if the fraction of sensitive revertants was larger than this error. Populations that show phenotypic sensitivity in all are in green, when just a fraction of the population is sensitive it is marked as yellow. Phenotypic assays were performed by replica plating individual clones in the presence of either 100 μg/mL of Rifampicin, 100 μg/mL of Streptomycin or both of them together (see Materials and Methods).
(XLSX)

S3 Table MIC of rifampicin and streptomycin in the resistance genotypes harbouring compensatory mutations. The minimum inhibitory concentration (MIC) was assessed for each resistance genotype reconstructed with a compensatory mutation (either RpoC^Q1226K^, *nusE* or *nusG*). Neither of the genotypes harbouring compensatory mutations show a difference in MIC compared to their respective ancestral genotypes.
(XLSX)

S4 Table List of strains used in this work. (XLSX)

S5 Table List of oligonucleotides used in this work. (XLSX)

S6 Table Raw data for all the figures. (XLSX)

## Abbreviations

2SEM: twice the standard error of the mean
5′-UTR: 5′ untranslated region
CFP: cyan fluorescent protein
CI: confidence interval
GLMEM: generalized linear mixed effects model
HSD: honest significant difference
IS: insertion sequence
OBAMAv2: One Bi-Allelic Marker Algorithm
RNAP: RNA polymerase
RT-qPCR: quantitative real-time PCR
SD: standard deviation
SNP: single nucleotide polymorphism
YFP: yellow fluorescent protein
