# Supplementary figures and images for: Multidrug-resistant bacteria compensate for the epistasis between resistances
Source: PLoS Biol. 2017 Apr 18;15(4):e2001741. doi: 10.1371/journal.pbio.2001741 (PMC5395140; doi:10.1371/journal.pbio.2001741)

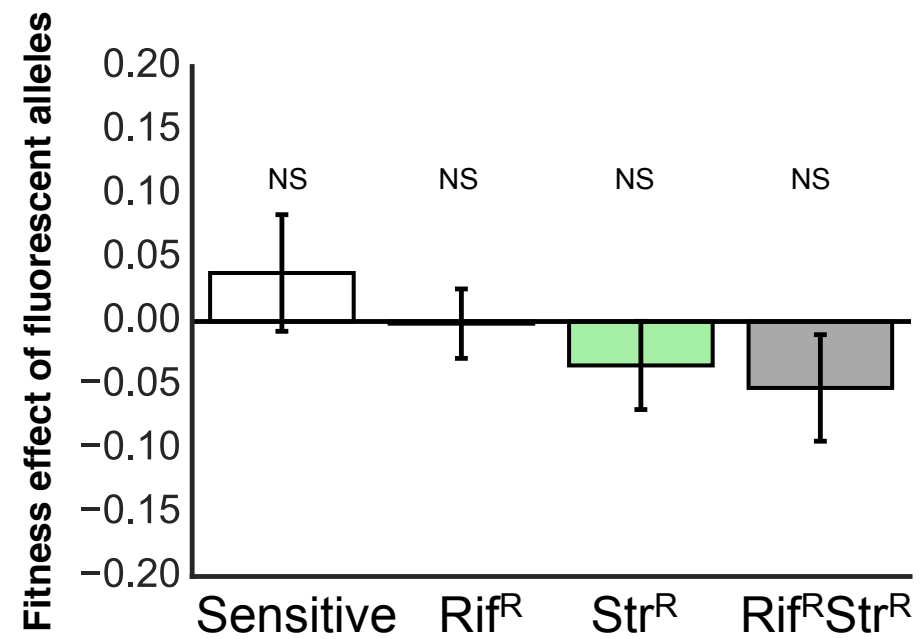

Supplement: S1 Fig — Each fluorescent background (both CFP and YFP) was competed against the same genotype without the fluorescence. NS indicates non-significance according to a one sample T-test, to test deviation from neutrality (Sensitive, s = 0.038 (2SEM = 0.046), P = 0.158; RifR, s = -0.002 (2SEM = 0.027), P = 0.867; StrR, s = -0.034 (2SEM = 0.035), P = 0.102; RifRStrR, s = -0.052, (2SEM = 0.042), P = 0.066). (PDF) [file pbio.2001741.s001.pdf]

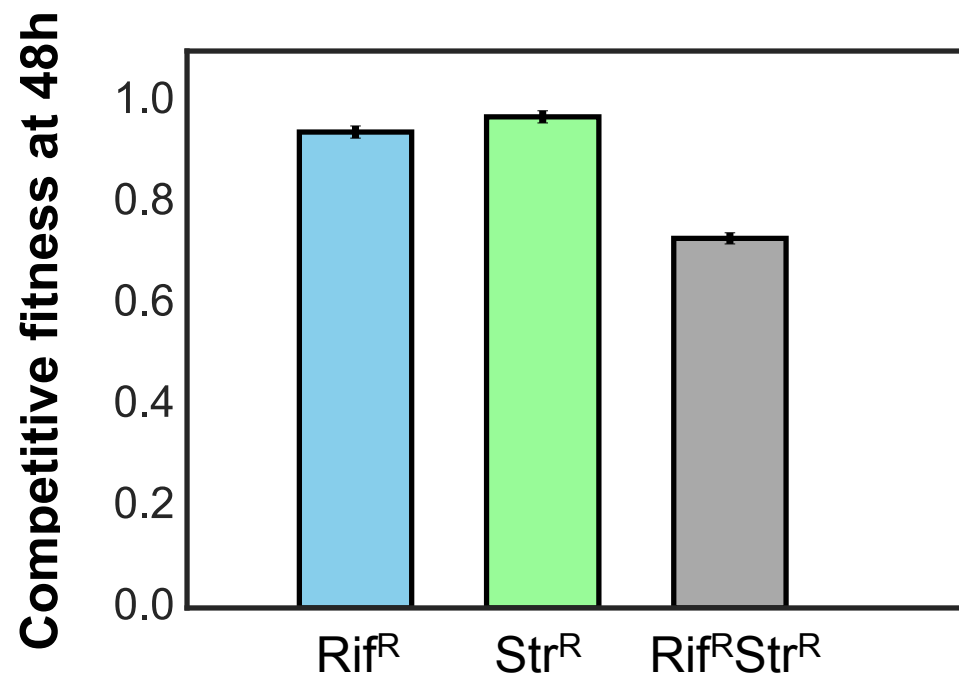

Supplement: S2 Fig — The RifR StrR background shows strong negative synergistic epistasis. Bars represent the mean fitness of the resistant strains when in competition with a sensitive, error bars represent 2SEM. (PDF) [file pbio.2001741.s002.pdf]

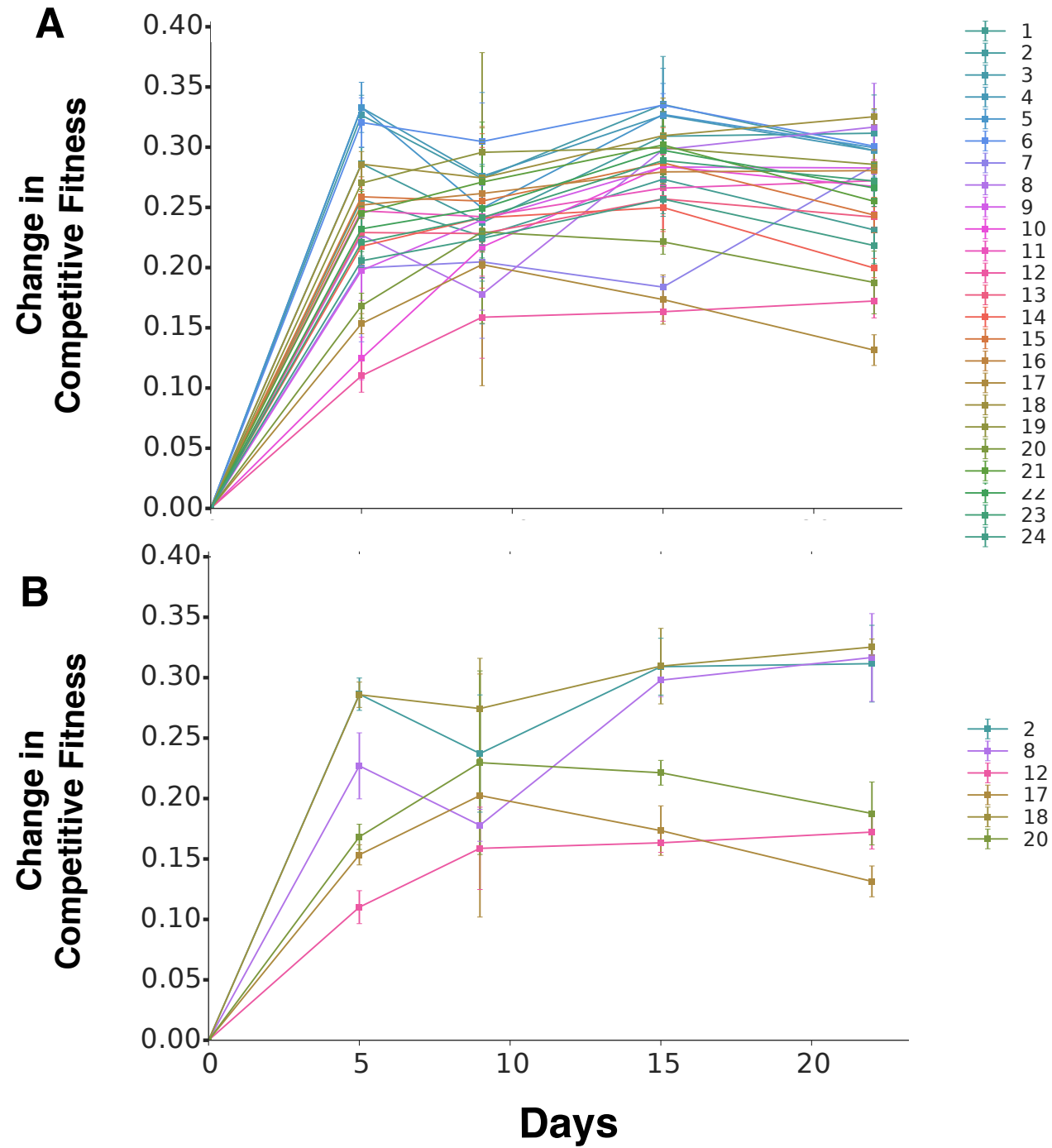

Supplement: S3 Fig — Error bars indicate the SEM across 3 independent replicates. In A are the dynamics of all the 24 populations, and in B are shown 6 indicative populations, for clarity. (PDF) [file pbio.2001741.s003.pdf]

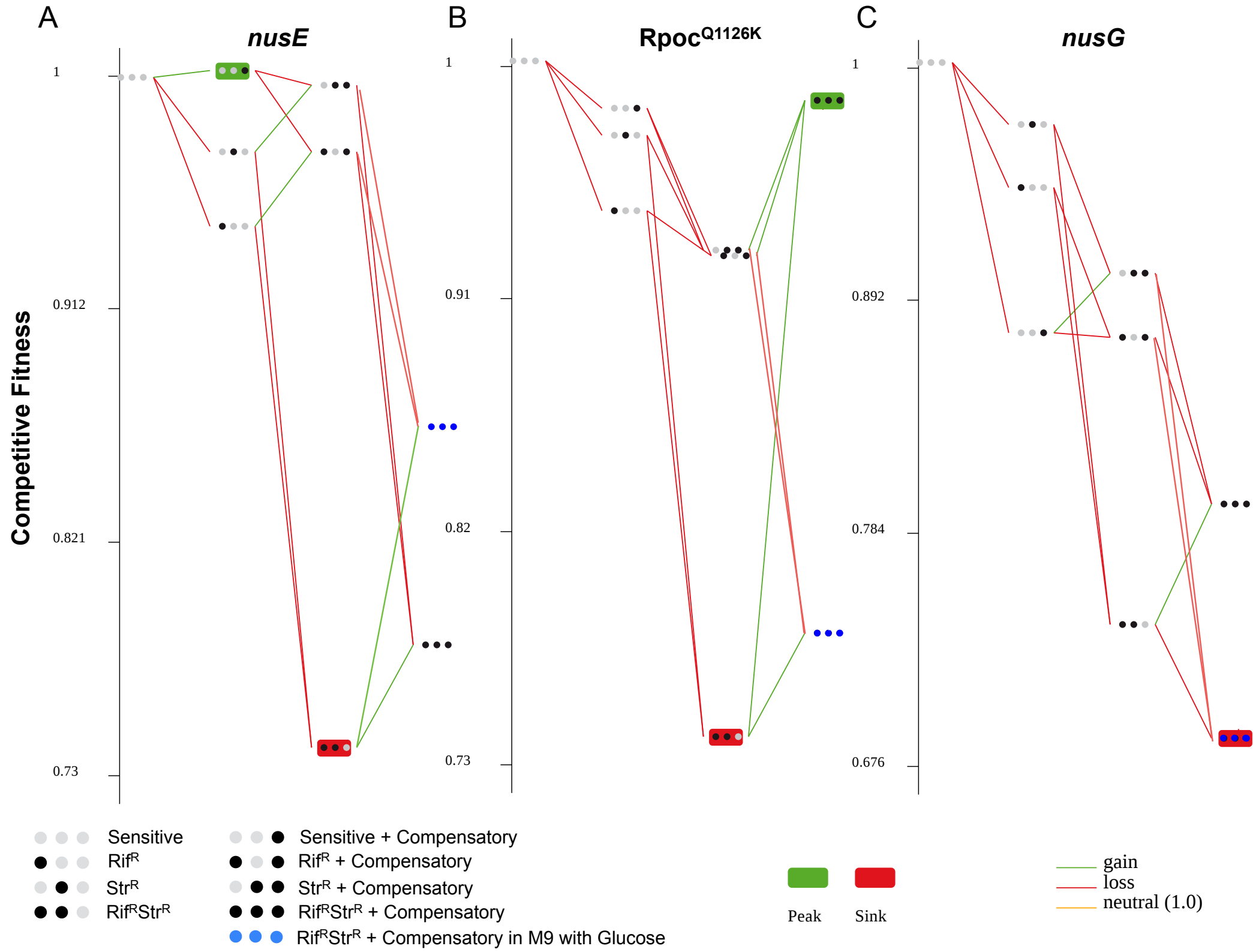

Supplement: S4 Fig — Genotypes are characterized by the presence (closed dots) or absence (open dots) of three different alleles, representing, respectively, the RifR mutation, the StrR mutation and one compensatory mutation, which changes depending on the panel. Peaks, in green, indicate the maximum fitness, and sings, in red indicate the genotype with the lowest fitness of each landscape of 3 alleles. Closed dots in blue represent the genotype in minimal medium supplemented with glucose. In A) nusE (third allele) improves the position in the landscape for all genotypes, since it is beneficial across all backgrounds. In B) and C), RpoCQ1226K and nusG, respectively, only improve the position of the double resistant genotype, with the magnitude of the improvement depending on the environment. Landscapes were constructed with the MAGELLAN software [73], and edited to include the genotypes in a different environments. (PDF) [file pbio.2001741.s004.pdf]

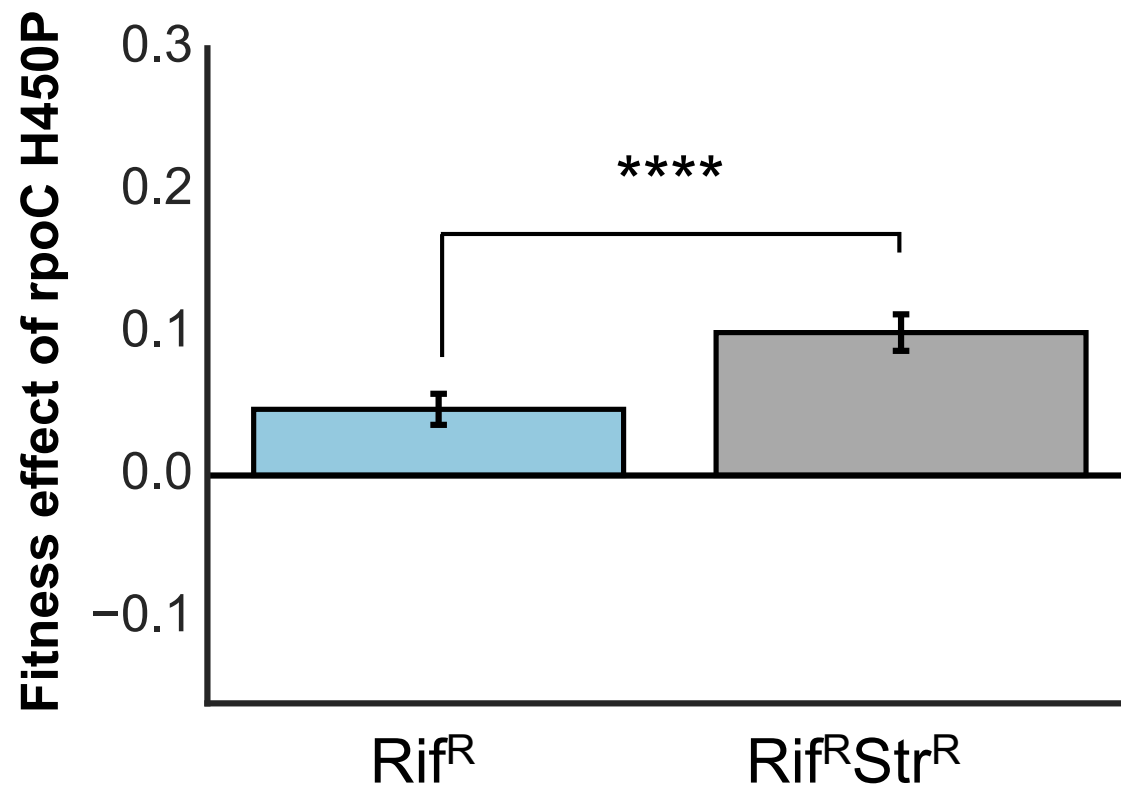

Supplement: S5 Fig — Mean effect on fitness (± 2 SEM) of the mutation in the single (in blue) and double resistant (in grey) backgrounds. (PDF) [file pbio.2001741.s005.pdf]

**A**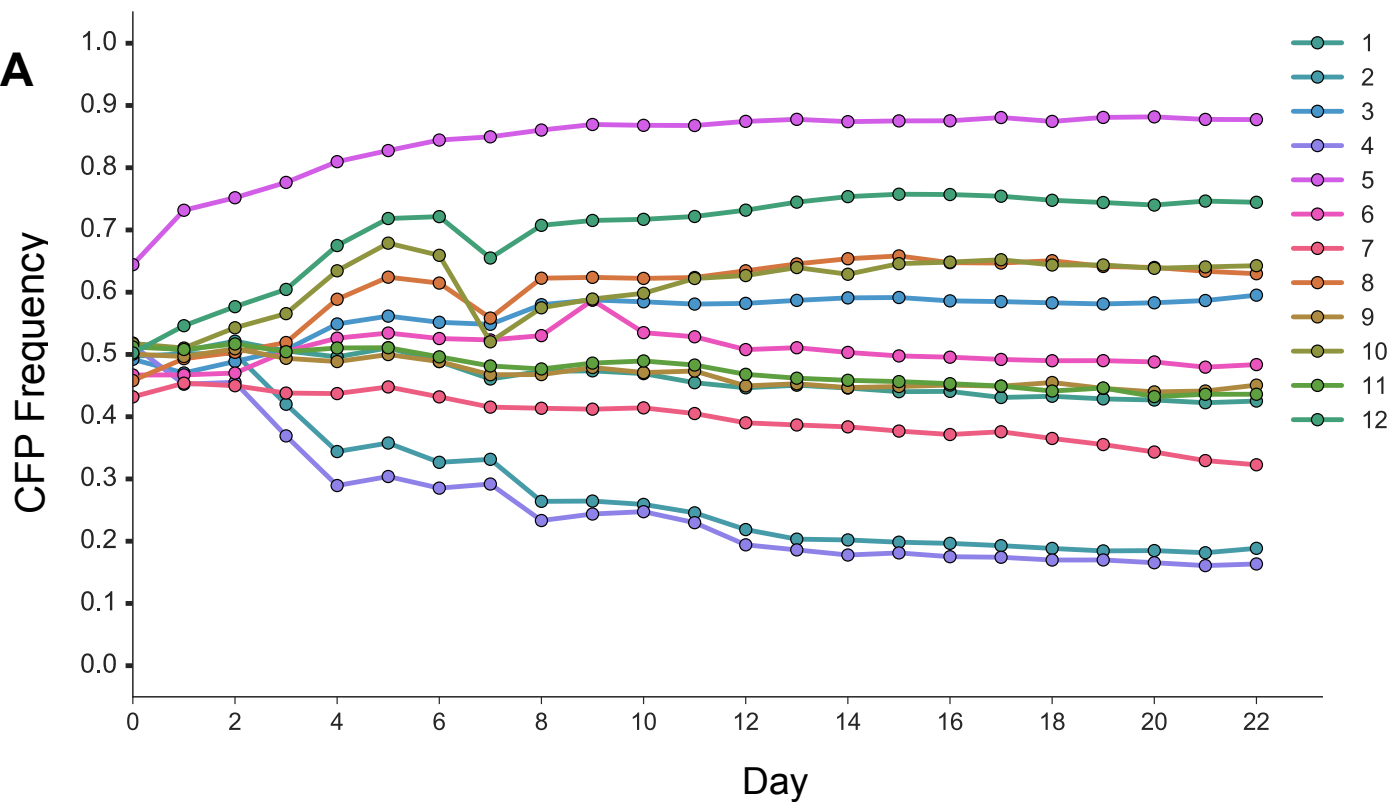**B**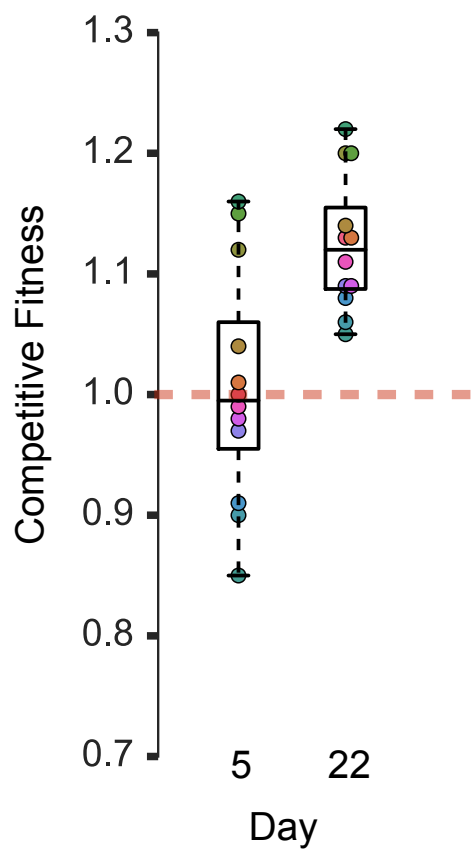**C**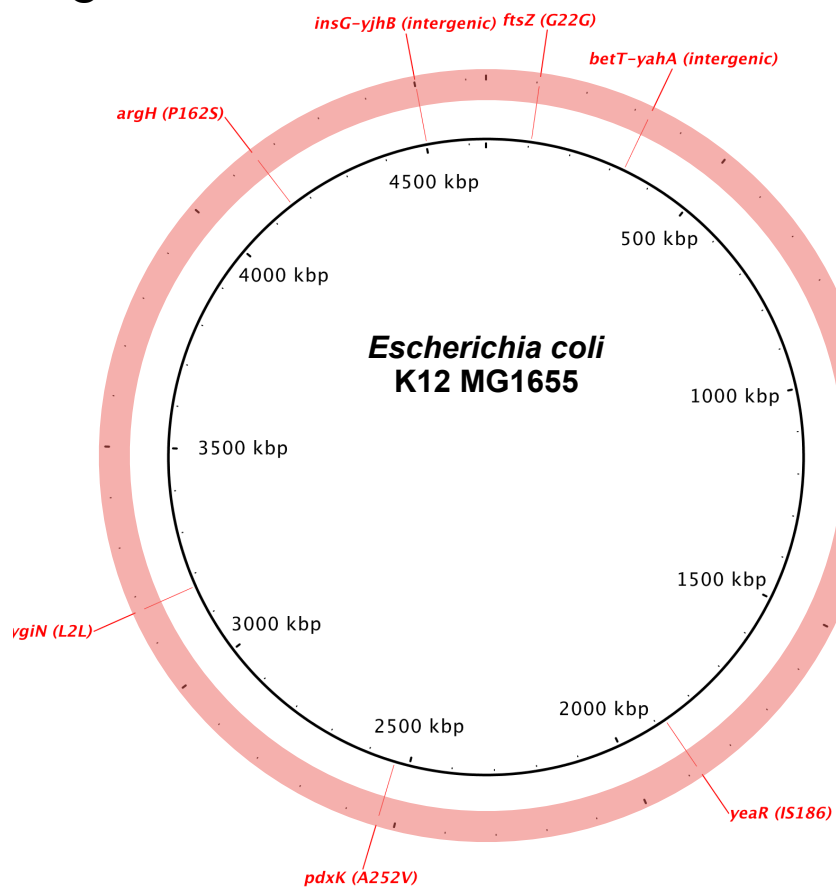

Supplement: S6 Fig — (A) Dynamics of a fluorescent neutral marker during propagation in rich media without antibiotics of 1:1 mixtures of YFP/CFP cells in 12 independent sensitive E. coli populations. (B) Competitive fitness of the sensitive evolving populations, at different days during adaptation. Each circle corresponds to a population with similar colour shown in panels A. Red dashed lines correspond to the competitive fitness of the sensitive strain. Box plots represent the median and quartiles Q2 and Q3, and whiskers show the last quartiles of the data. (C) Genetic basis of adaptation of the sensitive bacteria. Mutations identified through population sequencing in the evolved antibiotic sensitive background. Mutations detected at least in one population map at the position indicated in the circular plot (see also S1 Table). (PDF) [file pbio.2001741.s006.pdf]

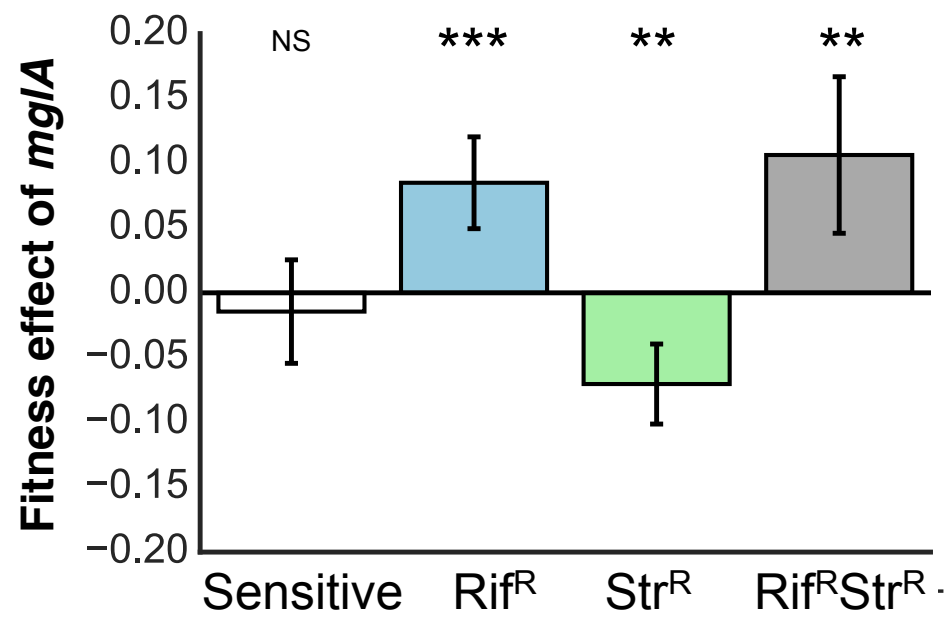

Supplement: S7 Fig — The knockout of mglA is beneficial in the RifR (s = 0.085 (2SEM = 0.035), P = 0.0006) and RifRStrR (s = 0.106 (2SEM = 0.031), P = 0.008) backgrounds, being neutral in the sensitive background (s = -0.014 (2SEM = 0.040), P = 0.483) and deleterious in the StrR background (s = -0.07 (2SEM = 0.031), P = 0.001). Bars indicate the mean effect on fitness and error bars indicate twice the SEM. (PDF) [file pbio.2001741.s007.pdf]

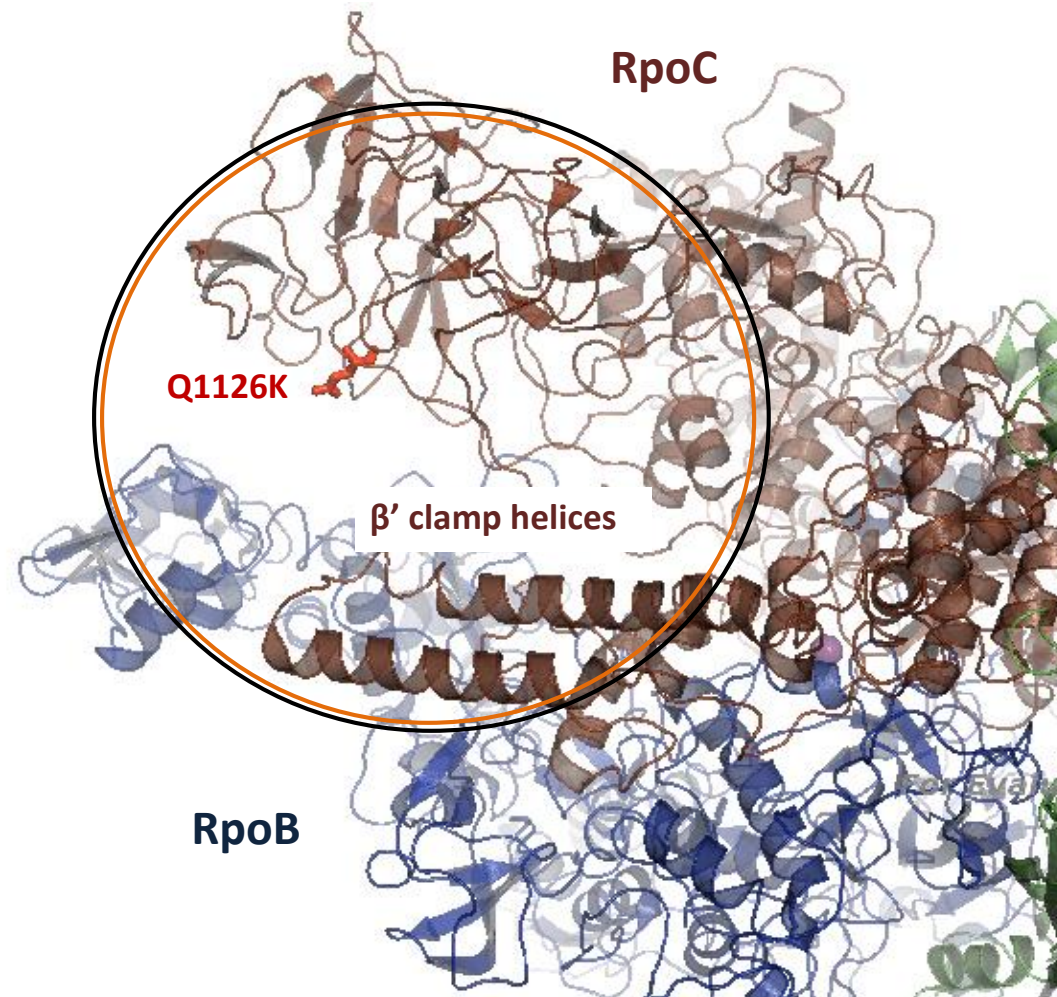

Supplement: S8 Fig — Detail of RNA polymerase showing the β (in blue) and β’ (in brown) subunits encoded by the rpoB and the rpoC genes, respectively. The proposed binding area of NusG N-terminal domain [75–77] is highlighted with a black circle and it partially overlaps with the DNA binding domain (highlighted with an orange circle). The β’ clamp helices are crucial for the NusG interaction with RpoC.Residue RpoCQ1126K (in red) is localized in a DNA binding region domain and maps closely to the proposed binding area of NusG. Figure drawn in Pymol using the crystal structure of E. coli RNAP (PDB 3LUO). (PDF) [file pbio.2001741.s008.pdf]
